# Identification of Novel Natural Dual HDAC and Hsp90 Inhibitors for Metastatic TNBC Using e-Pharmacophore Modeling, Molecular Docking, and Molecular Dynamics Studies

**DOI:** 10.3390/molecules28041771

**Published:** 2023-02-13

**Authors:** Nihal AbdElmoniem, Marwa H. Abdallah, Rua M. Mukhtar, Fatima Moutasim, Ahmed Rafie Ahmed, Alaa Edris, Walaa Ibraheem, Alaa A. Makki, Eman M. Elshamly, Rashid Elhag, Wadah Osman, Ramzi A. Mothana, Abdulrahim A. Alzain

**Affiliations:** 1 Department of Pharmaceutical Chemistry, Faculty of Pharmacy, University of Gezira, Wad Madani 21111, Sudan; 2 Department of Molecular Biotechnology, Hochschule Anhalt, 06846 Dessau-Roßlau, Germany; 3 Department of Biology, College of Science and Technology, Florida A & M University, Tallahassee, FL 32307, USA; 4 Department of Pharmacognosy, Faculty of Pharmacy, University of Khartoum, Khartoum 11114, Sudan; 5 Department of Pharmacognosy, College of Pharmacy, King Saud University, Riyadh 11451, Saudi Arabia

**Keywords:** cancer, HDAC6 and Hsp90, natural products, docking, molecular dynamics, ADMET

## Abstract

Breast cancer (BC) is one of the main types of cancer that endangers women’s lives. The characteristics of triple-negative breast cancer (TNBC) include a high rate of recurrence and the capacity for metastasis; therefore, new therapies are urgently needed to combat TNBC. Dual targeting HDAC6 and Hsp90 has shown good synergistic effects in treating metastatic TNBC. The goal of this study was to find potential HDAC6 and Hsp90 dual inhibitors. Therefore, several in silico approaches have been used. An e-pharmacophore model generation based on the HDAC6-ligand complex and subsequently a pharmacophore-based virtual screening on 270,450 natural compounds from the ZINC were performed, which resulted in 12,663 compounds that corresponded to the obtained pharmacophoric hypothesis. These compounds were docked into HDAC6 and Hsp90. This resulted in the identification of three compounds with good docking scores and favorable free binding energy against the two targets. The top three compounds, namely ZINC000096116556, ZINC000020761262, and ZINC000217668954, were further subjected to ADME prediction and molecular dynamic simulations, which showed promising results in terms of pharmacokinetic properties and stability. As a result, these three compounds can be considered potential HDAC6 and Hsp90 dual inhibitors and are recommended for experimental evaluation.

## 1. Introduction

Breast cancer (BC) is the most common malignancy in women and the second leading cause of cancer mortality in women [1]. Despite the significant developments in cancer treatment, the American Cancer Society (ACS) predicted 284,200 new BC cases and 44,130 deaths in 2021 [2]. In terms of clinical and biological behavior, BC is a very complex, heterogeneous disease with multiple categories [3,4]. It can be classified according to the expression of proteins and genes into Three categories: hormone receptor-positive (HR+), human epidermal growth factor receptor 2 (HER2)-enriched, and triple-negative breast cancer (TNBC) [5]. TNBC is an aggressive and dangerous of all BC types. TNBC is characterized by the absence of the progesterone receptor (PR), estrogen receptor (ER), and HER2 expression and amplification, and it accounts for approximately 15% of all BC cases [6,7]. Compared to all other subsets of BC, TNBC malignancies have a much higher recurrence rate with a high probability of metastasis [8,9]. Accordingly, the average length of stay of patients with significant metastatic TNBC is eighteen months [10]. Therefore, new targeted drugs that improve TNBC therapy outcomes and minimize patient mortality are urgently needed [11].

Bevacizumab, a monoclonal anti-vascular endothelial growth factor (VEGF-A) antibody, is currently the only targeted drug authorized for metastatic BC treatment, but it is ineffective in the treatment of metastatic TNBC subsets [12]. As a result, there is a desperate need to create new goals and methods to treat tumors of this type [13]. In this term, both histone deacetylase (HDAC6) and heat shock protein (Hsp90) are potential therapeutic targets for cancer therapy [14].

According to their homology, the histone deacetylase (HDAC) family is divided into four groups (I, II, III, and IV), with isoforms classified into yeast histone deacetylases and cell location [15]. The main functions of HDAC are to control gene expression and cellular signaling by modifying histones and non-histone proteins by removing acetyl groups attached to lysine residues [16]. The HDAC6 isoform is a class IIB cytoplasmic lysine deacetylase that regulates the acetylation of non-histone proteins such as transcription factors (FOXP3 and p53), Hsp90, cortactin, and tubulin [17]. HDAC regulates several processes that are considered to have a role in the formation of TNBC, such as stem cell self-renewal and proliferation or epithelial-mesenchymal transition (EMT), which promotes metastasis and cancer cell invasion [18]. Moreover, previous research has demonstrated that HDAC6 is a key regulator of cell migration [19,20,21] and an excellent target for therapeutic development against BC metastasis [22]. Recent investigations, however, have revealed that inhibiting HDAC6 alone has no substantial cytotoxicity; hence, this may be a promising target for synergistic therapy with additional medicines such as Hsp90 inhibitors [23]. Because HDAC6 controls the deacetylation of acetyl-Hsp90, inhibiting it would result in the acetylation of Hsp90 and the loss of its chaperone function [24,25].

HSP90 is a type of molecular chaperone that is highly conserved between species. The Hsp90 chaperone function enables appropriate folding and conformational stability for oncogenic client proteins, such as steroid receptors, tyrosine kinases, and cell cycle regulators, which are critical at all stages of BC tumor growth and metastasis [26]. High Hsp90 expression in BC patients, particularly those with TNBC, is linked to a worse prognosis and lower relapse-free survival [27]. As a result, in the early 1990s, Hsp90 was proposed as a target for cancer drugs [26].

Finding effective TNBC therapy will require a lot of work [28], and this process is time-consuming due to comprehensive clinical and preclinical safety testing [29]. Among the many methods of computational drug development, a drug structure design can be used to develop and optimize the chemical structure of compounds to make them suitable for clinical testing [30]. Using pharmacophore model generation, scientists can identify and analyze molecules before synthesizing them. As a result, the time and expense associated with medication research and development can be reduced [31], allowing for the assessment of biological activity in a large number of chemically diverse molecules and the discovery of new compounds as well as molecular poses that are responsible for the activity [32,33,34]. Pharmacophore modeling is a valuable tool for obtaining a better understanding of current data and generating more potent drugs [35].

Phytochemicals are natural bioactive components found in dietary and non-dietary plants that are becoming promising anticancer and antimetastatic drugs. Thus, determining the ability of phytochemicals that inhibit HDAC6 and Hsp90 activity may be a good way to prevent BC metastasis [36].

Given the importance of HDAC6 and Hsp90 in TNBC metastasis and the ongoing development of breast cancer-targeting drugs, this study was conducted to find novel dual HDAC6 and Hsp90 inhibitors using *e*-pharmacophore modeling, screening, molecular docking, and MM-GBSA calculations to calculate the binding affinity towards the two targets. In addition, the results were augmented using molecular dynamics (MD) simulations to investigate the stability of the compound–protein complexes.

## 2. Results and Discussion

**Figure 1:**
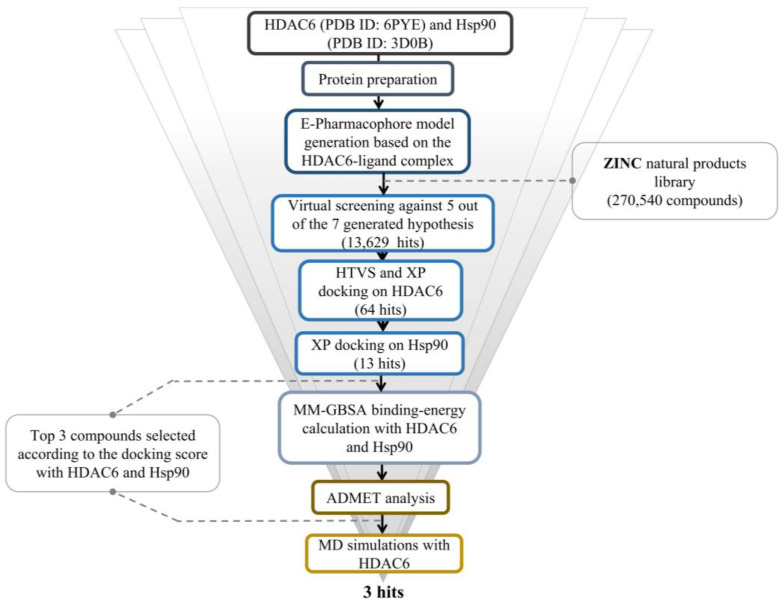
The overall work of the study.

Figure 1 summarizes the study’s workflow.

### 2.1. E-Pharmacophore Modeling and Screening

**Figure 2:**
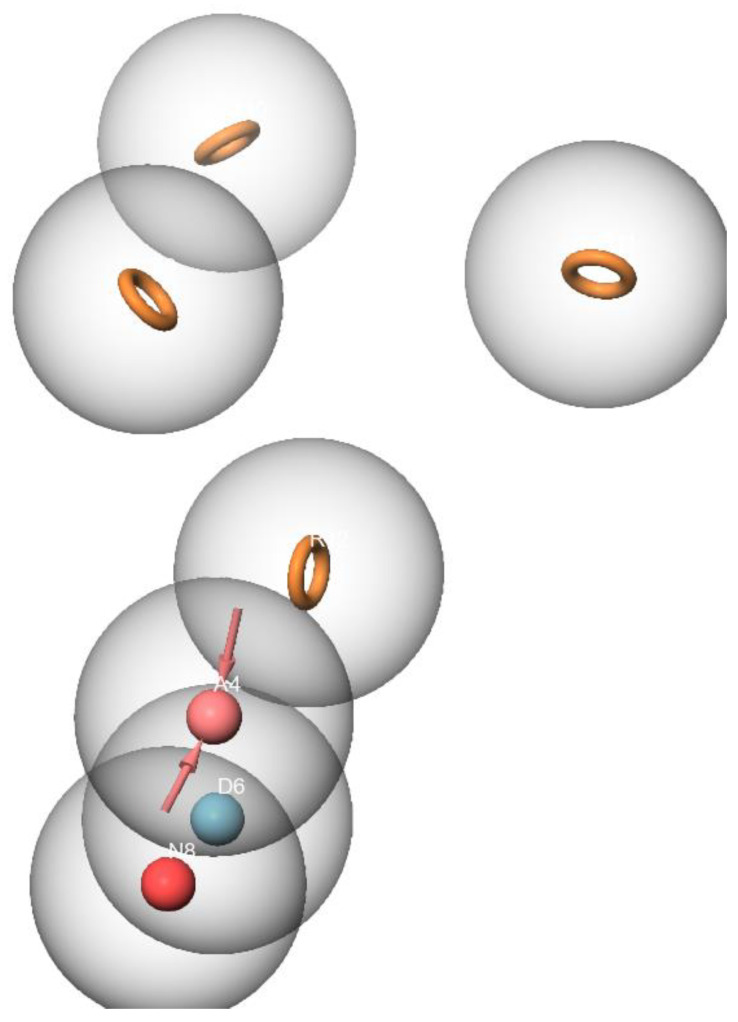
The pharmacophore hypothesis developed with HDAC6 (PDB ID: 6PYE) using the receptor -ligand complex. The hypothesis was generated using “Generate hypothesis from multiple ligands” option of Phase software. Pink sphere with arrow, hydrogen-bond acceptor (A); yellow open circle, aromatic ring (R); blue sphere with arrow, hydrogen-bond donor (D).

Numerous studies have demonstrated that inhibiting HDAC6 and Hsp90 simultaneously has a synergistic anticancer effect [37,38,39]. This study aimed to identify natural compounds targeting the HDAC6 and Hsp90 proteins using an in silico approach. A pharmacophoric hypothesis containing seven features (one donor (D), one acceptor (A), negative ionic, and four aromatic rings) was obtained using the Phase module of Maestro (Figure 2). These features were used to test a library comprising 270,540 natural chemicals against the pharmacophoric hypothesis. Of those natural chemicals, 13,629 compounds matched five out of seven pharmacophoric hypotheses and were thus selected for the upcoming molecular docking.

### 2.2. Molecular Docking and MM-GBSA

**Figure 3:**
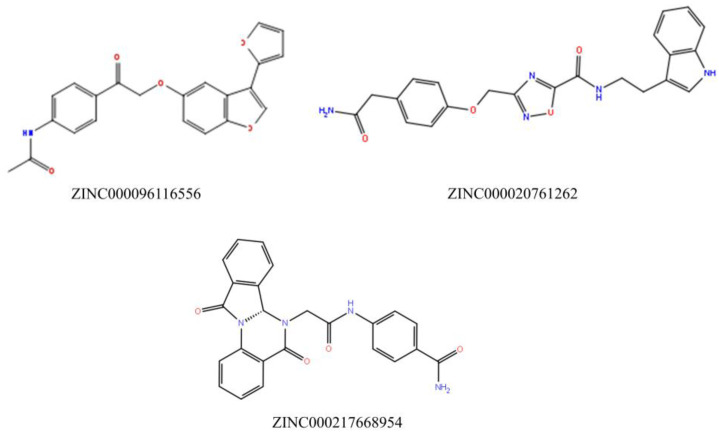
Top three hits with dual binding affinities toward HDAC6 and Hsp90.

**Figure 4:**
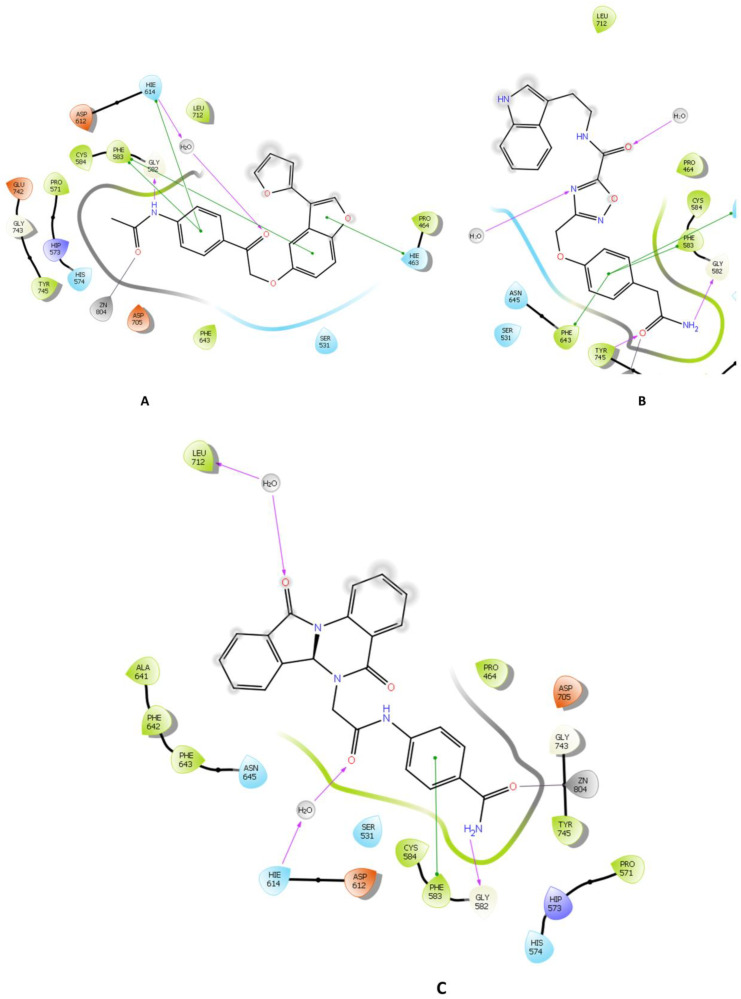
2D interaction of the top three compounds in complex with HDAC6 (PDB ID: 6PYE) using XP docking mode of Glide software. (**A**) compound A; (**B**) compound B; (**C**) compound C. The hydrogen-bond interactions with residues are represented by a purple dashed arrow directed towards the electron donor. The hydrophobic residues are shown in green.

**Figure 5:**
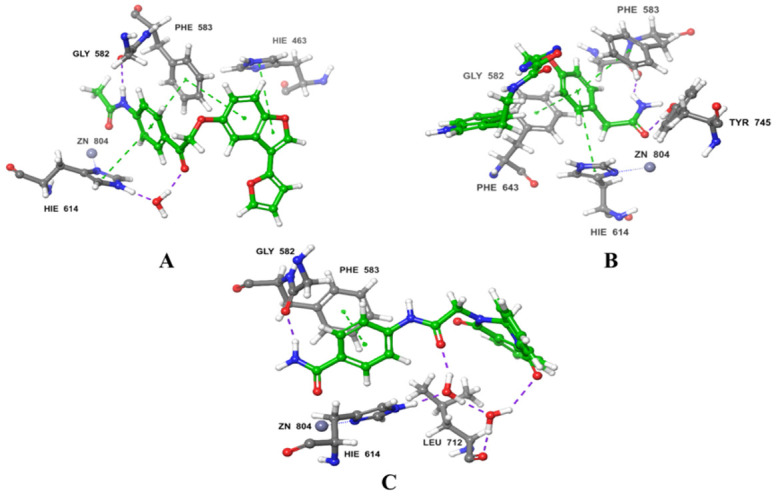
3D interaction diagrams of the three ligands (shown in green) with HDAC6 (PDB ID: 6PYE). (**A**) compound A; (**B**) compound B; (**C**) compound C. The hydrogen-bond interactions are represented by purple dashes, while the pi–pi stacking interactions are represented by orange dashes.

**Figure 6:**
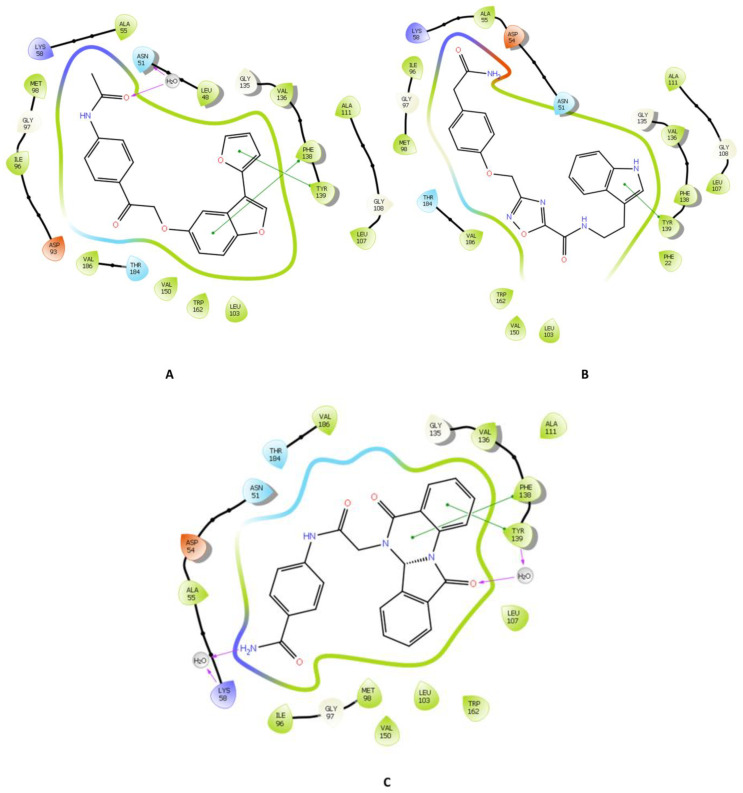
2D interaction of the top three compounds in complex with Hsp90 (ID: 3D0B) using XP docking mode of Glide software. (**A**) compound A; (**B**) compound B; (**C**) compound C. The hydrogen-bond interactions with residues are represented by a purple dashed arrow directed towards the electron donor. The hydrophobic residues are shown in green.

**Figure 7:**
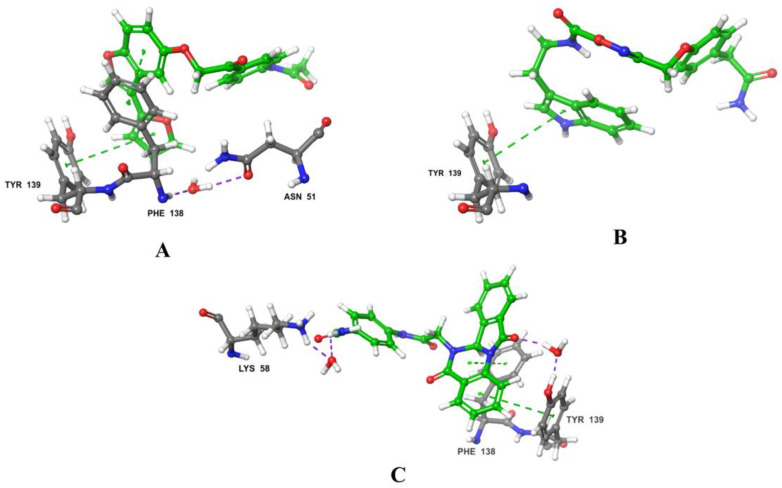
3D interaction diagrams of the three ligands (shown in green) with Hsp90. (**A**) compound A; (**B**) compound B; (**C**) compound C. The hydrogen-bond interactions are represented by purple dashes, while the pi–pi stacking interactions are represented by orange dashes.

Throughout the docking process, the 13,629 compounds were subjected to the HTVS mode of Glide against the HDAC6 protein. A total of 64 compounds showed docking scores better than the reference (<−8.782 Kcal/mol) and were then docked into Hsp90 through the XP docking mode. Thirteen compounds resulting from this XP had docking scores of ≤−6 Kcal/mol. The Prime module’s MM-GBSA calculation was applied to the three compounds (Figure 3) with the highest XP docking scores to determine the free binding energies to HDAC6 and Hsp90, as shown in Table 1. The three compounds showed favorable binding energies ranging from −42.45 to −23.31 Kcal/mol and from −52.69 to −24.66 Kcal/mol for HDAC6 and Hsp90, respectively. Co-crystallized ligands bound with HDAC6 protein showed lower docking scores of −6.14 kcal/mol. The top three compounds with satisfactory free binding energies and good docking scores for the two targets were further investigated, including visual examination of intermolecular interactions with HDAC6 (Table 2 and Figure 4 and Figure 5) and Hsp90 (Table 2 and Figure 6 and Figure 7).

ZINC000096116556, ZINC000020761262, and ZINC000217668954, labeled A-B-C, respectively, were the top-ranked compounds that achieved the highest docking scores in the library of natural products of the ZINC database with Hsp90 and satisfactory docking scores with HDAC6 (Figure 3). The interaction patterns between compounds A–C, HDAC6, and Hsp90 are presented in Figure 4, Figure 5, Figure 6 and Figure 7.

HDAC6 consists of a cap, a zinc-binding group (ZBG), and a linker that connects the first two components [40]. It belongs to the metal–protein family in which the stability of the catalytic center is crucially maintained by the zinc ion, and furthermore, the inhibitors of HDAC6 interact by chelating with this ion [41,42]. In this regard, compound A showed a metal coordination bond between its carbonyl oxygen, near the amide group, and the positively charged zinc ion. Moreover, the HDAC6-compound A complex showed three pi-cation interactions with HIE614, HIE463, and PHE583 residues and H-bonds with HIE614 residue (Figure 4A and Figure 5A). Furthermore, compound A displayed a hydrophilic interaction through a water bridge with HIE614. Compound A also exhibited hydrophobic interaction through CYS584, TYR745, PHE583, PRO571, PRO464, LEU712, and PHE643 residues. Similar binding interactions were observed with other inhibitors [43,44,45,46], among which was belinostat, an FDA-approved HDAC6 inhibitor [43,44]. Further investigations on belinostat has been performed by calculating its docking score which found to be −9.511 which is lower than that of compound A (−9.968). Moreover, binding free energy (BFE) has been computed using MM-GBSA which revealed that compound A has BFE of −42.45 Kcal/mol which is higher than the reference belinostat (with a BFE value of −32.77 Kcal/mol).

Benzamide is a ZBG that is important in determining the potency of HDAC6 inhibitors [40]. Compound B contains the benzamide group as a ZBG; it showed interaction through the carbonyl group. Compound B showed three types of interactions with the binding pocket of HDAC6 which are two H-bonds with GLY582 and TYR745 residues; pi–pi interactions through PHE643, PHE583, and HIE614; and hydrophobic interactions with the PHE643, TYR745, PRO571, PHE583, CYS584, PRO464, and LEU712 amino residues (Figure 4B and Figure 5B). HDAC6 binding affinity and van der Waals interactions are enhanced by pointing to the Phe643 and Leu 712 residues in the so-called L1-loop pocket, as studied by Bai [47].

The carbonyl group of the ZBG benzamide of compound C interacts with the zinc ion, which contributes to the stability of the complex. Moreover, compound C and HDAC6 interactions include three pi–pi interactions through PHE583, while hydrogen bonds were displayed with HIE614, LEU712, and GLY582 residues (Figure 4C and Figure 5C). It also showed hydrophilic interactions through water bridges with HIE614 and LEU712. Leu712 is thought to be crucial for inhibitory actions according to Nguyen’s research [44]. Furthermore, hydrophobic interactions through ALA641, PRO464, CYS584, TYR745, PHE643, PHE583, PRO571, PHE642, and LEU712 residues were noticed. Two residues (PHE583 and LEU712) were reported by H. Losson et al. and were considered important residues for the inhibition of HDAC6, which were also with compound C [43].

It is essential to emphasize that the HDAC6 catalytic domain, where the acetylated lysine is deacetylated, contains a typical narrow hydrophobic channel formed by residues PRO464, GLY582, PHE583, PHE643, and LEU712 [48]. Interactions with one or more residues in the catalytic site were observed with compounds A, B, and C. An interaction with PHE643 was reported with *R*-trichostatin (TSA), a histone deacetylase inhibitor [49]. In this regard, compound B formed a hydrogen bond with TYR745 which emphasized bond stabilizes the reaction intermediate to eventually release the product with deacetylated lysine residues. This interaction was observed with the bioactive conformer of TSA, but it was absent in the inactive one [49].

On the other hand, the three compounds interacted with Hsp90’s essential amino acid residues. For example, the Hsp90 compound A complex conveyed a pi-cation interaction through PHE138 and TYR139. PHE138 was found to be involved in the inhibition of Hsp90 by the Sanchez group [50]. Furthermore, it formed a hydrophilic interaction through a water bridge with ASN51. Asn51 was discovered to be an essential residue to interact stably with Hsp90, as reported by Magwenyane [51]. The hydrophobic interactions were noticed with ALA111, LEU107, LEU103, VAL150, TYR139, PHE138, TRP162, VAL136, VAL186, MET98, ILE96, ALA55, and LEU48 residues (Figure 6A and 7A). M. Abbasi et al., who reported these findings, highlighted the significance of interactions with PHE138, ALA55, LEU107, VAL186, TYR139, and VAL136, in addition to other amino acid residues [52,53].

Regarding compound B interactions with Hsp90, TYR139 residue was involved in a pi–pi interaction, which was also described by Gewirth et al. [53]. Hydrophobic interactions were seen with PHE22, LEU107, VAL136, ALA111, VAL150, LEU103, VAL186, MET98, ILE96, ALA55, TYR139, PHE138, and TRP162 residues. Interactions with ILE96, ALA55, PHE138, MET98, and LEU107 residues were also documented by Rampogu et al. [54] as crucial residues that inhibit the ATP-binding activity of Hsp90 (Figure 6B and 7B).

Many interactions were formed between compound C and the Hsp90 binding site (Figure 6C and Figure 7C). Pi–pi interactions with PHE138 and TYR139 residues which have been revealed to play key role in their complex formation were also observed by Rezvani et al. [55]. Moreover, LYS58 and TYR139 made H-bond interactions, whereas TRP162, LEU103, TYR139, PHE138, LEU107, VAL150, ALA55, MET98, ILE96, VAL186, VAL136, and ALA111 residues were observed in hydrophobic interactions. Additionally, compound C showed hydrophilic interactions through water bridges with LYS58 and TYR139, and these two residues were also reported by Jia [56]. PHE138 had been documented by El-Shafey et al. [57] as one of the residues crucial to Hsp90’s active binding site’s recognition of their reference ligand, geldanamycin.

The top three compounds, ZINC000096116556, ZINC000020761262, and ZINC000217668954, were further subjected to ADME prediction and MD simulations.

### 2.3. ADMET Prediction

The pharmacokinetic profile of the effective clinical candidates comprises its ADMET properties (absorption, distribution, metabolism, elimination, and toxicity), which are crucial for assessing their pharmacodynamics effect; therefore, these molecules must pass the ADMET test to be considered drug-like [54]. In this study, ADMET analysis was conducted on the three best-found hits with higher molecular docking and free binding energy using the QikProp module of Maestro (Table 3). According to their ADMET profile, these compounds passed “the rule of 5” and other pharmacokinetic tests with a (0) value, indicating that they were drug-like in agreement with their ADMET properties. Pharmacokinetic parameters such as QPPCaco and QPlogBB show the likelihood of transporting a molecule through the gut and the brain–blood barrier, respectively, thus allowing the prediction of the compound’s permeation through several barrier models. ZINC000096116556 has a greater gastrointestinal absorption, with a QPPCaco value of 1480.139, compared to ZINC000217668954, ZINC000020761262, and the reference, which have values of 105.569, 21.522, and 100.046, respectively. Limited CNS penetration is predicted by low QPlogBB values of (−0.618, −1.797, −2.276) for ZINC000096116556, ZINC000217668954, and ZINC000020761262, respectively, which are almost in the same range as the reference value of (−2.123). These top three hits have conformational independent aqueous values of (CIQplogs; −5.547, −4.819, −4.679), indicating their good solubility in accordance with their aqueous solubility (QPlogS; −5.141, −4.479, −2.254), which affects how drugs are absorbed and distributed across the body. Other ADMET parameters, such as QPlogPo/w values, showed an acceptable lipophilicity profile of the three compounds, ZINC000035424559, ZINC000085569484, and ZINC000015674312, with values of 2.068, 2.292, 1.258, respectively.

Regarding Percent Human-Oral Absorption, none of the top three compounds deviates from the recommended values (0 to 100%). ZINC000096116556 has a greater HPOA of (100%) in comparison with ZINC000217668954, ZINC000020761262, and the reference HPOA, which have values of 71.807, 56.718, and 69.793, respectively. This property fully agrees with human oral absorption values since both measure the same parameter. None of them had a low HOA value (HOA values coded 1, 2, or 3 for low, medium, and high, respectively). Furthermore, our findings revealed that the QPlog HERG value of ZINC000020761262 was much lower than the other compounds and the reference compound. This demonstrated that the reference compound could be more cardiotoxic than our hits (Table 3).

### 2.4. Molecular Dynamic (MD)

**Figure 8:**
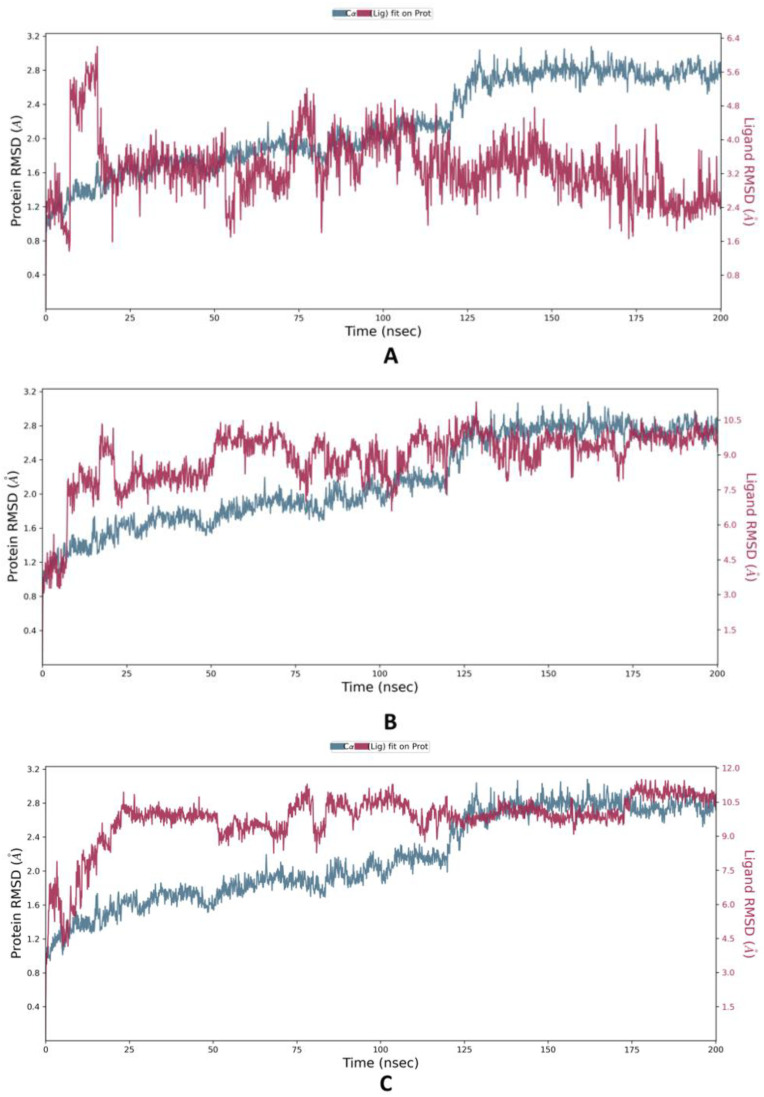
The protein–ligand RMSD plot of the top three compounds complexed with HDAC6 (PDB ID: 6PYE) during 200 ns molecular dynamics simulation using Desmond software. (**A**) compound A; (**B**) compound B; (**C**) compound C. Here, Y-axis represented the RMSD values of ligands and protein in molecular distance unit Angstrom (A), while X-axis demonstrated the time in nanoseconds (nsec).

In order to assess the flexibility and stability of the protein HDAC–Ligands (ZINC000096116556, ZINC000020761262, ZINC000217668954) complexes, molecular dynamics simulations for 200 ns were carried out using the Desmond software [56]. Such MD results included the root mean square deviation (RMSD), which was evaluated to confirm the stability of the protein system during the simulation period by measuring the variable distance between atoms where a distance range of 1–3 Å is generally acceptable for small globular proteins [57,58]. The HDAC–ZINC000096116556 complex fluctuated throughout the simulation with a maximum peak of less than 3 Å; however, it remained lower than the other ligands and even the protein backbone until it showed sudden stability in ~25 ns and remained bound until ~100; after that, the ligand showed significant fluctuations until the end of the simulation time. ZINC000020761262 and ZINC000217668954 exhibited similar fluctuation patterns, since they showed deviations until ~100 ns and maintained steadiness until 200 ns, with little deviations as observed in Figure 8.

**Figure 9:**
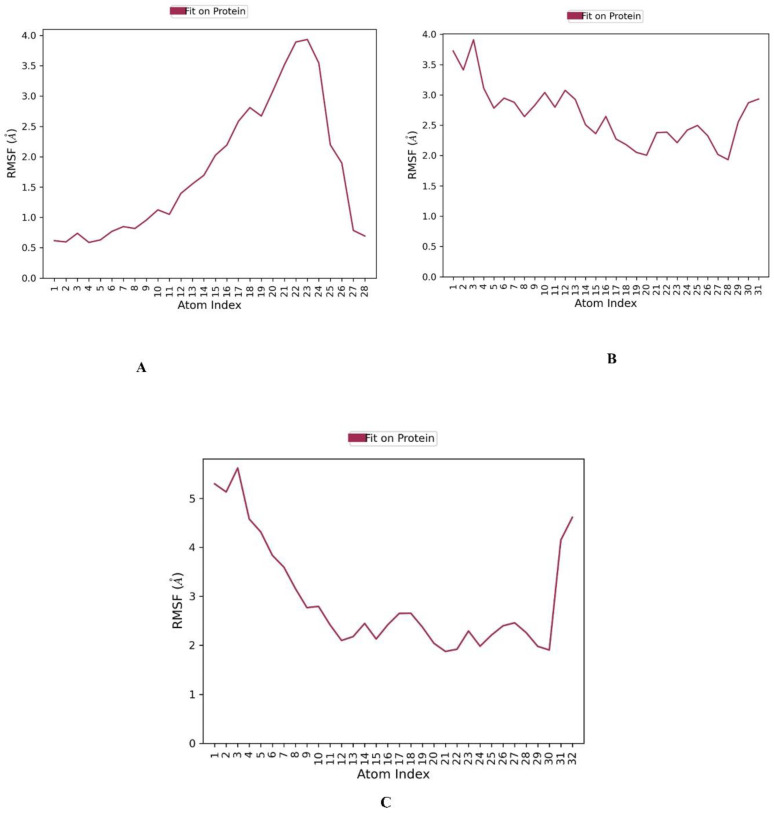
The ligand RMSF plot of the top three compounds complexed with HDAC6 (PDB ID: 6PYE) during 200 ns molecular dynamics simulation using Desmond software. (**A**) compound A; (**B**) compound B; (**C**) compound C. Here, Y axis represented the RMSF values in molecular distance unit Angstrom (A), while X axis demonstrated atom number of the ligand.

The RMSF (root mean square fluctuation) of each residue can be used to assess which residues are involved in protein or complex changes in structure. Low RMSF residues are more stable since the motions of the residue atoms during modeling are limited, and vice versa [59,60], ZINC000096116556 bound to HDAC6 has a notably low RMSF average value of 2.04 Å, indicating its interaction stability as illustrated in (Figure 9). By contrast, ZINC000020761262 and ZINC000217668954 have relatively high values of 4.89 and 3.38 Å respectively.

**Figure 10:**
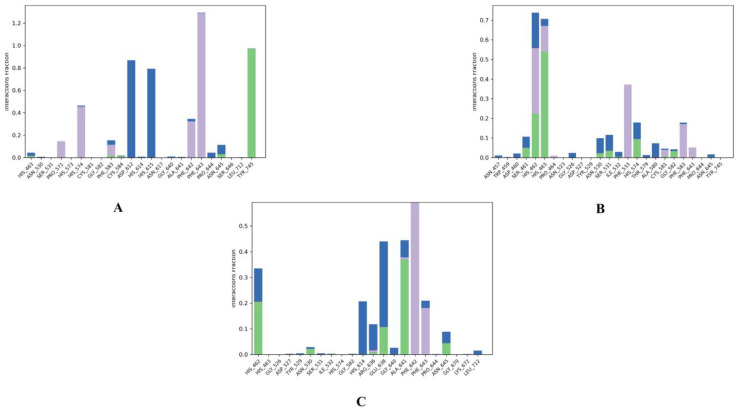
Protein–ligand contact histogram of the top three compounds complexed with HDAC6 (PDB ID: 6PYE) during 200 ns molecular dynamics simulation using Desmond software. (**A**) compound A; (**B**) compound B; (**C**) compound C. Here, X-axis represented the residues and Y-axis represented the interactions fraction. Different colours in the histogram represented different types of bond interactions fraction. The green colour represented H-bond, violet represented hydrophobic, pink represented ionic, and blue represented water bridge interaction.

Generally, protein-ligand interactions are facilitated by hydrogen bonds, and their strength in a water environment (Figure 10 and Figure S1 Supplementary Materials) provides further provisions for the docking results for HDAC6 and ZINC000096116556, ZINC000020761262, ZINC000217668954 interactions. ZINC000096116556 formed strong hydrophobic interactions with PHE643 (130%), HIS574 (~48%), and PHE642 (~38%). It also had hydrogen bonds with TYR745 (~100%) and water bridge interactions with HIS615 (78%) and ASP612 (88%). ZINC000020761262 interacted through water bridge bonds with HIS462 (25%); in addition, it formed hydrophobic interactions with PHE533 (48%), HIS462 (30%), PHE583 (19%), and HIS463 (11%) and formed hydrogen bonds with HIS463 (53%), HIS462 (23%), and HIS574 (10%). ZINC000217668954 primarily interacted with PHE642 (>50%) through hydrophobic bonds and formed hydrogen bonds with GLU638 (13%), ALA641 (38%), and HIS462 (21%). Through water bridge bonds, meanwhile, it formed water bridge interactions with HIS614 (35%), GLU638 (22%), HIS462 (13%), and ALA641 (<10%). These interactions confirm the docking results, since they show the same type of interactions with (PHE 643) and (PHE583) through hydrophobic bonds and with ALA641 through the water bridge bond.

As shown in Figure S2 (Supplementary Materials), the calculated ligand parameters for the top three compounds included RMSD, which for the three ligands stabilized at 0.9659, 2.406, and 1.701 A˚, respectively, throughout the simulation period, demonstrating the better stability of ZINC000096116556 compared to the other ligands. Moreover, the stabilities of the complexes were examined using the radius of gyrus (Rg) measurement to determine the compactness. The most stable ligands were those with the lowest Rg value. It can be seen from Table 4 that the average Rg values obtained from the complexes HDAC- ZINC000096116556 complex, HDAC- ZINC000020761262 complex, and HDAC- ZINC000217668954 complex were about 5.630, 5.990, and 5.294 Å, respectively, indicating that the HDAC-ZINC000217668954 complex has best compactness compared to the other complexes. Meanwhile, the SASA analysis was carried out to complete the stability analysis of each hit ligand complex, and the identification of SASA was conducted to anticipate the protein conformational changes that water molecules may access throughout the simulation. Commonly, the stability of the ligand–receptor complex increases with decreasing SASA values. As shown in Table 4, the HDAC–ZINC000096116556 complex has the lowest SASA average value, i.e., 259 Å, compared to the other two hits. The molecular surface (MolSA) is equivalent to the Van der Waals surface zone, and the three compounds had values of 358.5, 429.5, and 553.1 Å^2^, respectively. Intramolecular H-bonds display the number of intramolecular hydrogen bonds in ligand atoms. The three compounds showed no significant intramolecular H-bonds, according to molecular dynamics data. Furthermore, each protein-ligand’s polar surface area (PSA), which is defined as the total number of polar atoms on all molecule surfaces, was measured. Less occupied space makes the complex more stable. ZINC000096116556 had a PSA average of 130 Å^2^. The simulation process for the three molecules also revealed the torsional trend of rotatable bonds (Figure S3, Supplementary File). As a result, each rotatable bond in these three compounds maintains its orientation during a simulation that lasts 200 nanoseconds, proving again the stability of their conformation.

## 3. Materials and Methods

In silico studies were conducted in Maestro v12.8 of the Schrödinger suite. Molecular dynamics simulations were performed using academic Desmond v6.5 by D.E. Shaw Research. 

### 3.1. Preparation of Proteins and Ligands

The crystallographic structures of HDAC6 and Hsp90 with PDB IDs 6PYE and 3D0B, respectively, were retrieved from the protein data bank (https://www.rcsb.org/ accessed on 25 May 2022). After that, with the Protein Preparation Wizard tool of Maestro [61], the two proteins’ structures were prepared for the upcoming procedures. Firstly, they underwent structural pre-processing where bond orders were assigned, absent hydrogen atoms were added, zero-order bonds to metals and disulfide bonds were created, incomplete side chains and loops were filled, water molecules beyond 5 Å were deleted, and het states were generated at 7 ± 2 pH via Epik tool. Secondly, the pre-processed structures underwent refinement. In this step, the crystallized water molecules’ orientations were assigned and the residues’ protonation states were checked and corrected via PROPKA. Finally, the refined structures were passed through a restrained minimization process under the OPLS4 force field, in which heavy atoms converged to an RMSD of 0.30 Å.

A library containing 270,540 natural product structures was obtained from the ZINC database (https://zinc.docking.org/ accessed on 25 May 2022), then, using the MacroModel tool with the default parameters [62], the library was subjected to energy minimization under the OPLS4 force field to ensure that the compounds’ structures were acceptable for further computational calculations.

### 3.2. E-pharmacophore Modeling and Virtual Screening

In the Phase module, the E-Pharmacophore generation panel was opened and the option “create a pharmacophore model using receptor-ligand complex” was selected to create a pharmacophore model from the HDAC6–ligand complex [63]. Subsequently, the prepared natural products’ library was screened against the generated pharmacophoric features via the Phase Ligand Screening panel, resulting in a group of compounds that matched the features [64].

### 3.3. Grid Generation and Molecular Docking

One of the main approaches in computational chemistry is molecular docking. Schrödinger’s Glide module includes three docking methodologies: HTVS (high-throughput virtual screening), SP (standard precision), and XP (extra precision). These three methodologies differ in terms of speed and accuracy [65,66].

To carry out molecular docking, the Receptor Grid Generation panel was used to create the grid boxes of the two proteins where the docking occurs [67]. Since the structures from which grids will be created are receptors with co-crystallized ligands, the co-crystallized ligands molecules were identified to be distinguished from the receptor. The van der Waals radius scaling settings were left as default: scaling factor = 1 and partial charge cutoff = 0.25. Then, the receptor grid generation process was allowed to run. Thereafter, the molecular docking was conducted using the Ligand Docking panel of the Glide module with the default settings (only the docking methodology was changed each time). The group of compounds obtained from the screening against the predicted pharmacophoric features was docked to the HDAC6-generated grid through the HTVS mode of Glide. The compounds that achieved high docking scores were further docked via the XP mode. In the end, the resulting compounds with high docking scores on HDAC6 were docked to the Hsp90 grid through the XP mode.

### 3.4. Free Binding Energy Calculations

The MM-GBSA panel of the Prime module was used to estimate the free binding energies of the top compounds according to their docking scores with HDAC6 and Hsp90. [68]. The free binding energy of the receptor–ligand complexes is determined by the following equation:

∆E = E_c_ − E_R_ − E_L_

where ∆E is the free binding energy, E_c_ is the target/ligand complex energy, E_R_ is the receptor energy, and E_L_ is the ligand energy [61]. The solvation model was set to be VSGB and the force field was OPLS4.

### 3.5. ADMET Analysis

The top compounds according to the docking score were also analyzed to predict their pharmacokinetic properties by the QikProp tool of Schrödinger [62]. This initial prediction may be useful in decreasing the failure probability in the future stages of drug development.

### 3.6. Molecular Dynamics (MD) Simulations

After ADME analysis, the top compounds bound to HDAC6 were subjected to the MD simulations utilizing the Desmond software to further investigate the binding interactions and stability of the receptor–ligand complexes [69]. Before starting the simulation process, it is necessary to install a suitable biological system by using the system builder panel, in which the compounds were immersed in 11663 TIP3P molecules in an orthorhombic figured box with diameters of 10 × 10 × 10 Å. Salt was added in a specific concentration of Na^+^ and Cl^−^ charge: 59.239 mM (Total charge + 38) for Na^+^ and 51.445 mM (Total charge-33) for Cl^−^. Then, the system underwent energy minimization using OPLS4. The system was equilibrated in a two-phase sequential equilibration simulation: isothermal-isochoric (NVT) and isothermal-isobaric (NPT) ensembles. After that, the simulation process was allowed to run for 200 ns using an NPT ensemble at a temperature of 1 K and pressure of 1 bar which was kept constant throughout the simulation duration. The Nose–Hoover chain and the Martyna–Tobias–Klein methods were used as a thermostat and a barostat, respectively. The cutoff radius = 9.0 Å. The trajectory of the recording interval was every 100 p. A total of 2000 frames were obtained during the simulation. The Simulation Interaction Diagram tool of Desmond was used to analyze the resulting frames, such as RMSD, RMSF, and receptor–ligand contacts. The RMSD for a frame X is calculated by the following equation (Equation (1)):

(1) $${\mathrm{RMSD}}_{x}=\sqrt{\frac{1}{N}\sum_{i=1}^{N}(r_{i}^{\prime }\left(t_{x}\right))-r_{i}\left(t_{ref}\right){)}^{2}\;}$$

where *N* is the number of atoms in the atom selection; *t_ref_* is the reference time (typically the first frame is used as the reference and it is regarded as time *t* = 0); and *r′* is the position of the selected atoms in a frame *x* after superimposing on the reference frame, where frame *x* is recorded at time *t_x_*. The procedure is repeated for every frame in the simulation trajectory.

The RMSF for a residue *i* is calculated by the following formula (Equation (2)):

(2) $${\mathrm{RMSF}}_{i}=\sqrt{\frac{1}{T}\sum_{t=1}^{T}<(r_{i}^{\prime }\left(t_{x}\right))-r_{i}\left(t_{ref}\right){)}^{2}>}$$

where *T* is the trajectory time over which the RMSF is calculated; *r_i_* is the position of residue I; *r*′ is the position of atoms in residue *i* after superposition on the reference; and the angle brackets indicate that the average of the squared distance is taken over the selection of atoms in the residue.

## 4. Conclusions

This study aimed to identify new dual inhibitors to combat one of the most aggressive types of breast cancer, TNBC, by considering two therapeutic targets: HDAC6 and Hsp90. Several computational chemistry methods, including e-pharmacophore modeling, molecular docking, MM-GBSA calculations, ADMET analysis, and molecular dynamics, were applied to screen 270,450 natural compounds from the ZINC database for their dual inhibition capability on the two targets. In this study, e- pharmacophore-based virtual screening followed by molecular docking have identified three compounds (ZINC000096116556, ZINC000020761262, and ZINC000217668954) that exhibited the highest docking scores with Hsp90 and satisfactory docking scores with HDAC6. In addition, the investigation of the interaction patterns of the three compounds showed many favorable interactions that agree with previous studies in the literature. Among these interactions, the chelation of the zinc ion in the HDAC6 active site plays a major role in HDAC6 inhibition. Further analysis of the three compounds revealed that they have acceptable free binding energies, favorable ADMET properties, and good interaction stabilities. These three hits may be potential inhibitors against HDAC6 and Hsp90, and if experimentally examined, they may serve as promising hits against TNBC in the near future.

## Figures and Tables

**Table 1:** Docking scores and MM-GBSA results of the top three compounds and the two reference ligands.

Name	HDAC6		Hsp90	
	XP Docking Score (Kcal/mol)	MM-GBSA dG Bind (Kcal/mol)	XP Docking Score (Kcal/mol)	MM-GBSA dG Bind (Kcal/mol)
HDAC6 bound ligand Hsp90 bound ligand ZINC000096116556	−8.782 - −9.968	−6.14 - −42.45	- −15.002 −11.863	- −84.41 −52.69
ZINC000020761262 ZINC000217668954	−9.391 −8.977	−23.19 −23.31	−8.580 −9.207	−32.88 −24.66

**Table 2:** Intermolecular interactions of the top three molecules with HDAC6 and Hsp90.

			HDAC6	
Name	Pi–pi Interaction (Total)	Hydrogen Bond Interaction (Total)	Hydrophobic Interaction (Total)	Other Interactions (Total)
ZINC000096116556	HIE614, HIE463, PHE583 (3)	HIE614 (1)	CYS584, TYR745, PHE583, PRO571, PRO464, LEU712, PHE643, (7)	Polar interaction: HIS574,SER531, HIE463, HIE614 (4) Charged negative: ASP705, ASP612, GLU742 (3) Charged positive: HIP573 (1)
ZINC000020761262	PHE643, PHE583, HIE614 (3)	GLY582, TYR745, (2)	PHE643, TYR745, PRO571, PHE583, CYS584, PRO464, LEU712 (7)	Polar interaction: HIS574,SER531, HIE614,ASN645 Charged negative: ASP705 Charged positive: HIP573
			Hsp90	
ZINC000217668954	PHE583 (1)	HIE614, LEU712, GLY582 (3)	ALA641, PRO464, CYS584, TYR745, PHE643, PHE583, PRO571, PHE642, LEU712 (9)	Polar interaction: HIS574,SER531, HIE614,ASN645 (4) Charged negative: ASP705, ASP612 (2) Charged positive: HIP573 (1)
ZINC000096116556	PHE138 TYR139 (2)	ASN51 (1)	ALA111, LEU107, LEU103, VAL150, TYR139, PHE138, TRP162, VAL136, VAL186, MET98, ILE96, ALA55, LEU48 (13)	Polar interaction: THR184, ASN51 (2) Charged negative: ASP93 (1) Charged positive: LYS58 (1)
ZINC000020761262	TYR139 (1)	- (0)	PHE22, LEU107, VAL136, ALA111, VAL150, LEU103, VAL186, MET98, ILE96, ALA55, TYR139, PHE138, TRP162 (13)	Polar interaction: THR184, ASN51 (2) Charged negative: ASP54 (1) Charged positive: LYS58 (1)
ZINC000217668954	PHE138 TYR139 (2)	LYS58, TYR139 (2)	TRP162, LEU103, TYR139, PHE138, LEU107, VAL150, ALA55, MET98, ILE96, VAL186, VAL136, ALA111 (12)	Polar interaction: THR184, ASN51 (2) Charged negative: ASP54 (1) Charged positive: LYS58 (1)

**Table 3:** ADME analysis of the top three compounds using the QikProp tool.

Drug-Likeness/Predicted ADME Descriptors	QPlogPo/w	QPlogS	CIQPlogS	QPlogHERG	QPPCaco	QPlogBB	QPPMDCK	Human Oral Absorption	%Human Oral Absorption	Rule of Five
ZINC000096116556	3.779	−5.141	−5.547	−6.631	1480.139	−0.618	755.835	3	100	0
ZINC000217668954	1.476	−4.479	−4.819	−6.751	105.569	−1.797	43.542	3	71.807	0
ZINC000020761262	1.011	−2.254	−4.679	−3.945	21.522	−2.276	18.642	2	56.718	0
6PYE bound ligand	3.417	−5.978	−6.843	−7.348	100.046	−2.123	113.473	2	69.793	1

**Table 4:** Ligand properties of the top three compounds during 200 ns molecular dynamics simulations using the Desmond tool.

	ZINC000096116556	ZINC000217668954	ZINC000020761262
RMSD	0.9659 ± 0.3655	2.406 ± 0.3266	1.701 ± 0.2344
rGyr	5.630 ± 0.1830	5.990 ± 0.3141	5.294 ± 0.1081
SASA	259.0 ± 29.50	477.5 ± 38.48	553.1 ± 53.04
MolSA	358.5 ± 1.674	429.5 ± 2.826	411.9 ± 2.248
intraHB	0.0 ± 0.0	0.0004995 ± 0.02235	0.01499 ± 0.1215
PSA	130.1 ± 4.000	264.6 ± 6.270	237.8 ± 4.264

## Data Availability

The datasets generated during and/or analyzed during the current study are available from the corresponding author upon reasonable request. Schrödinger suite is a commercial software (www.schrodinger.com). Academic Desmond by D.E. Shaw Research is available at https://www.deshawresearch.com/, accessed on 8 April 2022.

## Supplementary Materials

The following supporting information can be downloaded at: https://www.mdpi.com/article/10.3390/molecules28041771/s1, Figure S1. A timeline representation of the interactions and contacts (H-bonds, Hydrophobic, Ionic, Water bridges) of the top three compounds complexed with HDAC6 (PDB ID: 6PYE) during 200 ns molecular dynamics simulation using Desmond software. (A) compound A, (B) compound B, and (C) compound C. Figure S2. Ligand properties of the top three compounds complexed with HDAC6 (PDB ID: 6PYE) during 200 ns molecular dynamics simulation using Desmond software. (A) compound A, (B) compound B, and (C) compound C. Figure S3. Ligand torsion Profile of the top three compounds complexed with HDAC6 (PDB ID: 6PYE) during 200 ns molecular dynamics simulation using Desmond software. The top panel shows the 2d schematic of a ligand with color-coded rotatable bonds. Each rotatable bond torsion is accompanied by a dial plot and bar plots of the same color. (A) compound A, (B) compound B, and (C) compound C.

## References

[1] Januškevičienė I., Petrikaitė V. (2019). Heterogeneity of breast cancer: The importance of interaction between different tumor cell populations. Life Sci..

[2] Siegel R.L., Miller K.D., Fuchs H.E., Jemal A. (2021). Cancer Statistics, 2021. CA. Cancer J. Clin..

[3] Al-mahmood S., Sapiezynski J., Garbuzenko O.B., Minko T. (2018). Metastatic and triple-negative breast cancer: Challenges and treatment options. Drug Deliv. Transl. Res..

[4] Ressler S., Mlineritsch B., Greil R. (2010). Triple negative breast cancer. Memo-Mag. Eur. Med. Oncol..

[5] Vagia E., Mahalingam D., Cristofanilli M. (2020). The landscape of targeted therapies in TNBC. Cancers.

[6] Mallipeddi H., Thyagarajan A., Sahu R.P. (2021). Implications of Withaferin-A for triple-negative breast cancer chemoprevention. Biomed. Pharmacother..

[7] Oner G., Altintas S., Canturk Z., Tjalma W., Verhoeven Y., Van Berckelaer C., Berneman Z., Peeters M., Pauwels P., van Dam P.A. (2020). Triple-negative breast cancer—Role of immunology: A systemic review. Breast J..

[8] Shahi S., Akwii R., Sajib S., Farshbaf M.J., Kallem R.R., Putnam W., Wang W., Zhang R., Alvina K., Trippier P.C. (2020). Design, synthesis and structure-activity relationship study of novel urea compounds as FGFR1 inhibitors to treat metastatic triple-negative breast cancer. Eur. J. Med. Chem..

[9] Deepak K.G.K., Vempati R., Nagaraju G.P., Dasari V.R., Nagini S., Rao D.N., Malla R.R. (2020). Tumor microenvironment: Challenges and opportunities in targeting metastasis of triple negative breast cancer. Pharmacol. Res..

[10] Swain S.M., Kim S.B., Cortés J., Ro J., Semiglazov V., Campone M., Ciruelos E., Ferrero J.M., Schneeweiss A., Knott A. (2013). Pertuzumab, trastuzumab, and docetaxel for HER2-positive metastatic breast cancer (CLEOPATRA study): Overall survival results from a randomised, double-blind, placebo-controlled, phase 3 study. Lancet Oncol..

[11] Alothaim T., Charbonneau M., Tang X. (2021). HDAC6 inhibitors sensitize non-mesenchymal triple-negative breast cancer cells to cysteine deprivation. Sci. Rep..

[12] Marmé F. (2015). Targeted Therapies in Triple-Negative Breast Cancer. Breast Care.

[13] Lin C.W., Zheng T., Grande G., Nanna A.R., Rader C., Lerner R.A. (2021). A new immunochemical strategy for triple-negative breast cancer therapy. Sci. Rep..

[14] Yu S., Cai X., Wu C., Liu Y., Zhang J., Gong X., Wang X. (2017). Targeting HSP90-HDAC6 Regulating Network Implicates Precision Treatment of Breast Cancer. Int. J. Biol. Sci..

[15] De Ruijter A.J.M., Van Gennip A.H., Caron H.N., Kemp S., Van Kuilenburg A.B.P. (2003). Histone deacetylases (HDACs): Characterization of the classical HDAC family. Biochem. J..

[16] Witt O., Deubzer H.E., Milde T., Oehme I. (2009). HDAC family: What are the cancer relevant targets?. Cancer Lett..

[17] Kaluza D., Kroll J., Gesierich S., Yao T., Boon R.A., Hergenreider E., Tjwa M., Ro L., Seto E., Augustin H.G. (2011). Class IIb HDAC6 regulates endothelial cell migration and angiogenesis by deacetylation of cortactin. EMBO J..

[18] Fedele P., Orlando L., Cinieri S. (2017). Targeting triple negative breast cancer with histone deacetylase inhibitors. Expert Opin. Investig. Drugs.

[19] Hsieh Y., Tu H., Pan S., Liou J., Yang C. (2019). BBA-Molecular Cell Research Anti-metastatic activity of MPT0G211, a novel HDAC6 inhibitor, in human breast cancer cells in vitro and in vivo. BBA-Mol. Cell Res..

[20] Kra O.H., Mahboobi S., Sellmer A. (2014). Drugging the HDAC6–HSP90 interplay in malignant cells. Trends Pharm. Sci..

[21] Hideshima T., Mazitschek R., Qi J., Mimura N., Tseng J.C., Kung A.L., Bradner J.E., Anderson K.C. (2017). HDAC6 inhibitor WT161 downregulates growth factor receptors in breast cancer. Oncotarget.

[22] Li Y., Quan J., Song H., Li D., Ma E., Wang Y., Ma C. (2021). Novel pyrrolo[2,1-c][1,4]benzodiazepine-3,11-dione (PBD) derivatives as selective HDAC6 inhibitors to suppress tumor metastasis and invasion in vitro and in vivo. Bioorg. Chem..

[23] Reßing N., Melf S., Osko J., Schöler A., Skerhut A.J., Borkhardt A., Hauer J., Kassack M.U., Christianson D.W., Bhatia S. (2020). Multicomponent synthesis, binding mode and structure-activity relationships of selective histone deacetylase 6 (HDAC6) inhibitors with bifurcated capping groups. J. Med. Chem..

[24] Scroggins B.T., Robzyk K., Wang D., Marcu M.G., Tsutsumi S., Beebe K., Cotter R.J., Felts S., Toft D., Karnitz L. (2007). Short Article An Acetylation Site in the Middle Domain of Hsp90 Regulates Chaperone Function. Mol. Cell.

[25] Meng Q., Chen X., Sun L. (2011). Carbamazepine promotes Her-2 protein degradation in breast cancer cells by modulating HDAC6 activity and acetylation of Hsp90. Mol. Cell. Biochem..

[26] Koca İ., Özgür A., Er M., Gümüş M., Coşkun K.A., Tutar Y. (2016). Design and synthesis of pyrimidinyl acyl thioureas as novel Hsp90 inhibitors in invasive ductal breast cancer and its bone metastasis. Eur. J. Med. Chem..

[27] Mumin N.H., Drobnitzky N., Patel A., Lourenco L.M., Cahill F.F., Jiang Y., Kong A., Ryan A.J. (2019). Overcoming acquired resistance to HSP90 inhibition by targeting JAK-STAT signalling in triple-negative breast cancer. BMC Cancer.

[28] Chang Y.H., Vuong C.K., Ngo N.H., Yamashita T., Ye X., Futamura Y., Fukushige M., Obata-Yasuoka M., Hamada H., Osaka M. (2022). Extracellular vesicles derived from Wharton’s Jelly mesenchymal stem cells inhibit the tumor environment via the miR-125b/HIF1α signaling pathway. Sci. Rep..

[29] Alzain A.A., Elbadwi F.A. (2021). Identification of novel TMPRSS2 inhibitors for COVID-19 using e-pharmacophore modelling, molecular docking, molecular dynamics and quantum mechanics studies. Inform. Med. Unlocked.

[30] Idris M.O., Yekeen A.A., Suleiman O., Durojaye O.A. (2020). Computer-aided screening for potential TMPRSS2 inhibitors: A combination of pharmacophore modeling, molecular docking and molecular dynamics simulation approaches. J. Biomol. Struct. Dyn..

[31] Bhadoriya K.S., Sharma M.C., Sharma S., Jain S.V., Avchar M.H. (2013). An approach to design potent anti-Alzheimer’ s agents by 3D-QSAR studies on fused 5, 6-bicyclic heterocycles as c -secretase modulators using kNN–MFA methodology. Arab. J. Chem..

[32] Bendix F., Wolber G., Seidel T. (2010). 3D Pharmacophore Elucidation and Virtual Screening Strategies for 3D pharmacophore- based virtual screening. Drug Discov. Today Technol..

[33] Amnerkar N.D., Bhusari K.P. (2010). Synthesis, anticonvulsant activity and 3D-QSAR study of some prop-2-eneamido and 1-acetyl-pyrazolin derivatives of aminobenzothiazole. Eur. J. Med. Chem..

[34] Bhadoriya K.S., Kumawat N.K., Bhavthankar S.V., Avchar M.H., Dhumal D.M., Patil S.D., Jain S.V. (2015). Exploring 2D and 3D QSARs of benzimidazole derivatives as transient receptor potential melastatin 8 (TRPM8) antagonists using MLR and kNN-MFA methodology. J. SAUDI Chem. Soc..

[35] Khedkar S., Malde A., Coutinho E., Srivastava S. (2007). Pharmacophore Modeling in Drug Discovery and Development: An Overview. Med. Chem..

[36] Priya V.S., Pradiba D., Aarthy M., Singh K., Achary A., Vasanthi M. (2020). In-silico strategies for identification of potent inhibitor for MMP-1 to prevent metastasis of breast cancer. J. Biomol. Struct. Dyn..

[37] Bonanni D., Citarella A., Moi D., Pinzi L., Bergamini E., Rastelli G. (2021). Dual Targeting Strategies on Histone Deacetylase 6 (HDAC6) and Heat Shock Protein 90 (Hsp90). Curr. Med. Chem..

[38] Wu T.Y., Chen M., Chen I.C., Chen Y.J., Chen C.Y., Wang C.H., Cheng J.J., Nepali K., Chuang K.H., Liou J.P. Rational design of synthetically tractable HDAC6/HSP90 dual inhibitors to destroy immune-suppressive tumor microenvironment. J. Adv. Res..

[39] Wu Y.W., Chao M.W., Tu H.J., Chen L.C., Hsu K.C., Liou J.P., Yang C.R., Yen S.C., HuangFu W.C., Pan S.L. (2021). A novel dual HDAC and HSP90 inhibitor, MPT0G449, downregulates oncogenic pathways in human acute leukemia in vitro and in vivo. Oncogenesis.

[40] Zhang L., Zhang J., Jiang Q., Zhang L., Song W. (2018). Zinc binding groups for histone deacetylase inhibitors. J. Enzym. Inhib. Med. Chem..

[41] He J., Wang S., Liu X., Lin R., Deng F., Jia Z., Zhang C., Li Z., Zhu H., Tang L. (2020). Synthesis and Biological Evaluation of HDAC Inhibitors With a Novel Zinc Binding Group. Front. Chem..

[42] Lombardi P.M., Cole K.E., Dowling D.P., Christianson D.W. (2011). Structure, mechanism, and inhibition of histone deacetylases and related metalloenzymes. Curr. Opin. Struct. Biol..

[43] Losson H., Schnekenburger M., Dicato M., Diederich M. (2020). HDAC6—An Emerging Target Against Chronic. Cancers.

[44] Nguyen H.P., De Tran Q., Nguyen C.Q., Hoa T.P., Duy Binh T., Nhu Thao H., Hue B.T.B., Tuan N.T., Le Dang Q., Quoc Chau Thanh N. (2022). Anti-multiple myeloma potential of resynthesized belinostat derivatives: An experimental study on cytotoxic activity, drug combination, and docking studies. RSC Adv..

[45] Yao D., Jiang J., Zhang H., Huang Y., Huang J., Wang J. (2021). Bioorganic & Medicinal Chemistry Letters Design, synthesis and biological evaluation of dual mTOR/HDAC6 inhibitors in MDA-MB-231 cells. Bioorg. Med. Chem. Lett..

[46] Kassab S.E., Mowafy S., Alserw A.M., Seliem J.A., El-naggar S.M., Omar N.N., Awad M.M. (2019). Structure-based design generated novel hydroxamic acid based preferential HDAC6 lead inhibitor with on-target cytotoxic activity against primary choroid plexus carcinoma. J. Enzym. Inhib. Med. Chem..

[47] Bai P., Mondal P., Bagdasarian F.A., Rani N., Liu Y., Gomm A., Tocci D.R., Choi S.H., Wey H.Y., Tanzi R.E. (2022). Development of a potential PET probe for HDAC6 imaging in Alzheimer’s disease. Acta Pharm. Sin. B.

[48] Miyake Y., Keusch J.J., Wang L., Saito M., Hess D., Wang X., Melancon B.J., Helquist P., Gut H., Matthias P. (2016). Structural insights into HDAC6 tubulin deacetylation and its selective inhibition. Nat. Chem. Biol..

[49] Furumai R., Komatsu Y., Nishino N., Khochbin S., Yoshida M., Horinouchi S. (2001). Potent histone deacetylase inhibitors built from trichostatin A and cyclic tetrapeptide antibiotics including trapoxin. Proc. Natl. Acad. Sci. USA.

[50] Sanchez J., Carter T.R., Cohen M.S., Blagg B.S.J. (2020). Old and New Approaches to Target the Hsp90 Chaperone. Curr. Cancer Drug Targets.

[51] Magwenyane A.M., Ugbaja S.C., Amoako D.G., Somboro A.M., Khan R.B., Kumalo H.M. (2022). Heat Shock Protein 90 (HSP90) Inhibitors as Anticancer Medicines: A Review on the Computer-Aided Drug Discovery Approaches over the Past Five Years. Comput. Math. Methods Med..

[52] Abbasi M., Sadeghi-Aliabadi H., Amanlou M. (2017). Prediction of new Hsp90 inhibitors based on 3,4-isoxazolediamide scaffold using QSAR study, molecular docking and molecular dynamic simulation. DARU J. Pharm. Sci..

[53] Gewirth D.T. (2016). Paralog specific Hsp90 Inhibitors–a brief history and a bright future. Curr. Top. Med. Chem..

[54] Rampogu S., Parate S., Parameswaran S., Park C., Baek A., Son M., Park Y., Park S.J., Lee K.W. (2019). Natural compounds as potential Hsp90 inhibitors for breast cancer-Pharmacophore guided molecular modelling studies. Comput. Biol. Chem..

[55] Rezvani S., Ebadi A., Razzaghi-asl N. (2021). In silico identification of potential Hsp90 inhibitors via ensemble docking, DFT and molecular dynamics simulations. J. Biomol. Struct. Dyn..

[56] Jia J.M., Xu X.L., Liu F., Guo X.K., Zhang M.Y., Lu M.C., Xu L.L., Wei J.L., Zhu J., Zhang S.L. (2013). Identification, Design and Bio-Evaluation of Novel Hsp90 Inhibitors by Ligand-Based Virtual Screening. PLoS ONE.

[57] El-Shafey H.W., Gomaa R.M., El-Messery S.M., Goda F.E. (2020). Quinazoline Based HSP90 Inhibitors: Synthesis, Modeling Study and ADME Calculations Towards Breast Cancer Targeting. Bioorg. Med. Chem. Lett..

[58] Śledź P., Caflisch A. (2018). Protein structure-based drug design: From docking to molecular dynamics. Curr. Opin. Struct. Biol..

[59] Alzain A.A., Ismail A., Fadlelmola M., Mohamed M.A., Mahjoub M., Makki A.A., Elsaman T. (2022). De novo design of novel spike glycoprotein inhibitors using e-pharmacophore modeling, molecular hybridization, ADMET, quantum mechanics and molecular dynamics studies for COVID-19. Pak. J. Pharm. Sci..

[60] Martínez L. (2015). Automatic identification of mobile and rigid substructures in molecular dynamics simulations and fractional structural fluctuation analysis. PLoS ONE.

[61] Omer S.E., Ibrahim T.M., Krar O.A., Ali A.M., Makki A.A., Ibraheem W., Alzain A.A. (2022). Drug repurposing for SARS-CoV-2 main protease: Molecular docking and molecular dynamics investigations. Biochem. Biophys. Rep..

[62] Mohamed L.M., Eltigani M.M., Abdallah M.H., Ghaboosh H., Bin Jardan Y.A., Yusuf O., Elsaman T., Mohamed M.A., Alzain A.A. (2022). Discovery of novel natural products as dual MNK/PIM inhibitors for acute myeloid leukemia treatment: Pharmacophore modeling, molecular docking, and molecular dynamics studies. Front. Chem..

[63] Salam N.K., Nuti R., Sherman W. (2009). Novel method for generating structure-based pharmacophores using energetic analysis. J. Chem. Inf. Model..

[64] Dixon S.L., Smondyrev A.M., Knoll E.H., Rao S.N., Shaw D.E., Friesner R.A. (2006). PHASE: A new engine for pharmacophore perception, 3D QSAR model development, and 3D database screening: 1. Methodology and preliminary results. J. Comput. Aided. Mol. Des..

[65] Obubeid F.O., Eltigani M.M., Mukhtar R.M., Ibrahim R.A., Alzain M.A., Elbadawi F.A., Ghaboosh H., Alzain A.A. (2022). Dual targeting inhibitors for HIV-1 capsid and cyclophilin A: Molecular docking, molecular dynamics, and quantum mechanics. Mol. Simul..

[66] Friesner R.A., Murphy R.B., Repasky M.P., Frye L.L., Greenwood J.R., Halgren T.A., Sanschagrin P.C., Mainz D.T. (2006). Extra precision glide: Docking and scoring incorporating a model of hydrophobic enclosure for protein-ligand complexes. J. Med. Chem..

[67] Alzain A.A. (2022). Insights from computational studies on the potential of natural compounds as inhibitors against SARS-CoV-2 spike omicron variant. SAR QSAR Environ. Res..

[68] Suryanarayanan V., Singh S.K. (2015). Assessment of dual inhibition property of newly discovered inhibitors against PCAF and GCN5 through in silico screening, molecular dynamics simulation and DFT approach. J. Recept. Signal Transduct. Res..

[69] Baby K., Maity S., Mehta C.H., Suresh A., Nayak U.Y., Nayak Y. (2021). SARS-CoV-2 entry inhibitors by dual targeting TMPRSS2 and ACE2: An in silico drug repurposing study. Eur. J. Pharmacol..

