# Supplementary material for: Identification of Novel Natural Dual HDAC and Hsp90 Inhibitors for Metastatic TNBC Using e-Pharmacophore Modeling, Molecular Docking, and Molecular Dynamics Studies
Source: Molecules. 2023 Feb 13;28(4):1771. doi: 10.3390/molecules28041771 (PMC9965823; doi:10.3390/molecules28041771)
Supplement: Supplementary file 1 [file molecules-28-01771-s001.zip › molecules-2180733-supplementary.pdf]

molecules

Supplementary Materials

Identification of Novel Natural Dual HDAC and Hsp90 Inhibitors for Metastatic TNBC Using e-Pharmacophore Modeling, Molecular Docking, and Molecular Dynamics Studies

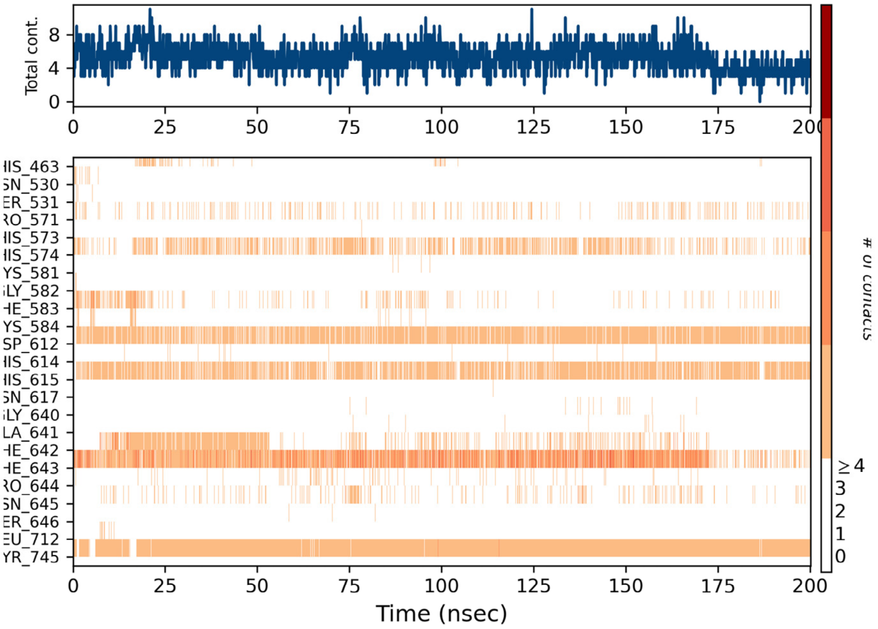

A

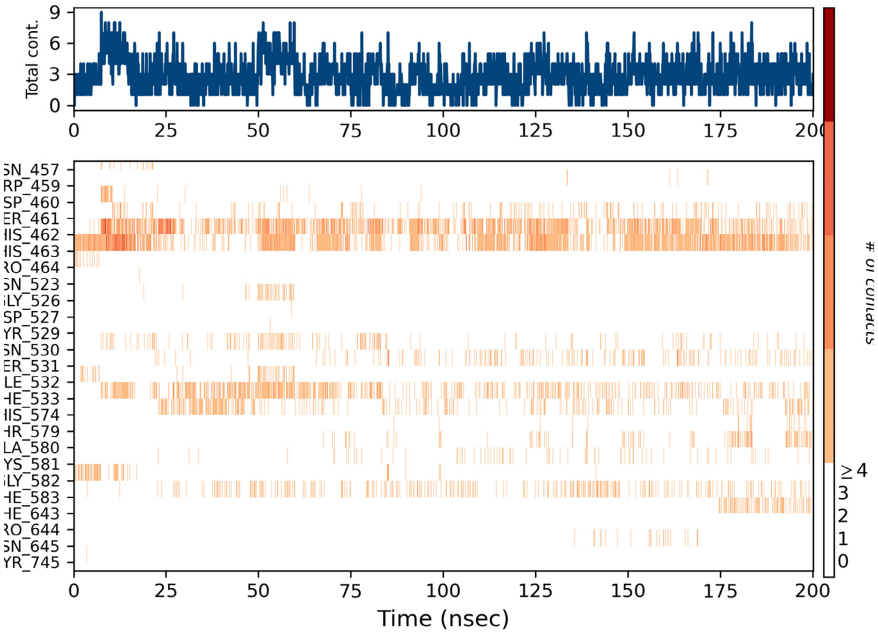

B

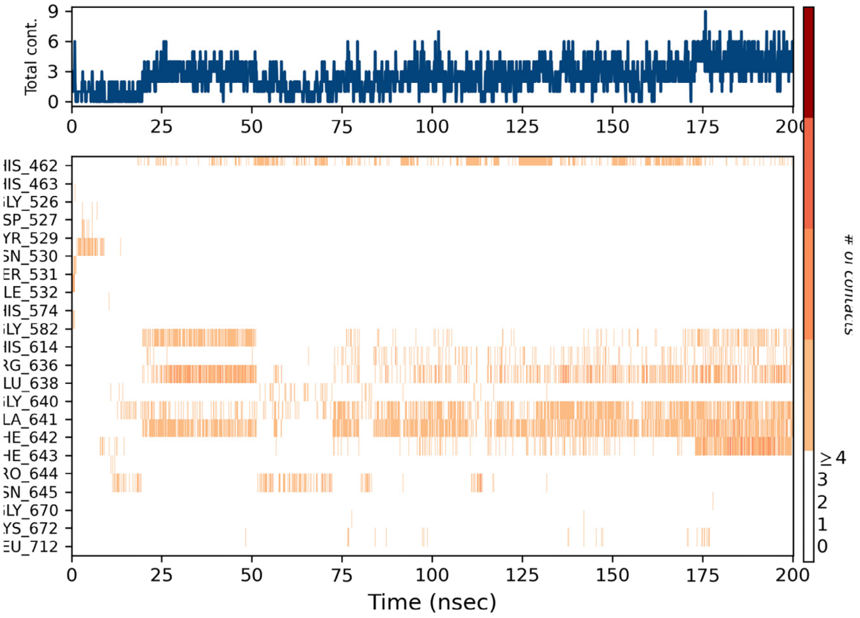

C

Figure S1. A timeline representation of the interactions and contacts (H-bonds, Hydrophobic, Ionic, Water bridges) of the top three compounds complexed with HDAC6 (PDB ID: 6PYE) during 200 ns molecular dynamics simulation using Desmond software. (A) compound A, (B) compound B, and (C) compound C.

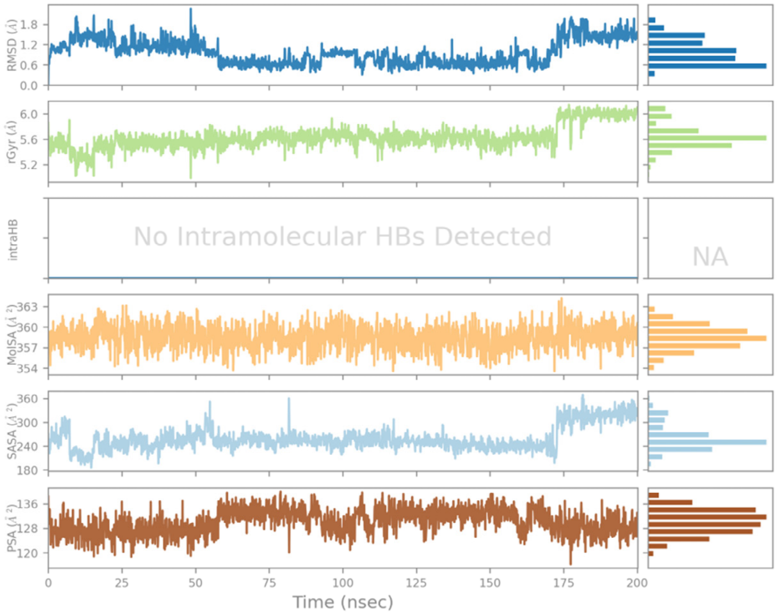
RMSD (Å)
0.0
0.6
1.2
1.8
rGyr (Å)
5.2
5.6
6.0
intraHB
No Intramolecular HBs Detected
NA
MolSA (Å²)
354
357
360
363
SASA (Å²)
180
240
300
360
PSA (Å²)
120
128
136
0
25
50
75
100
125
150
175
200
Time (nsec)

A

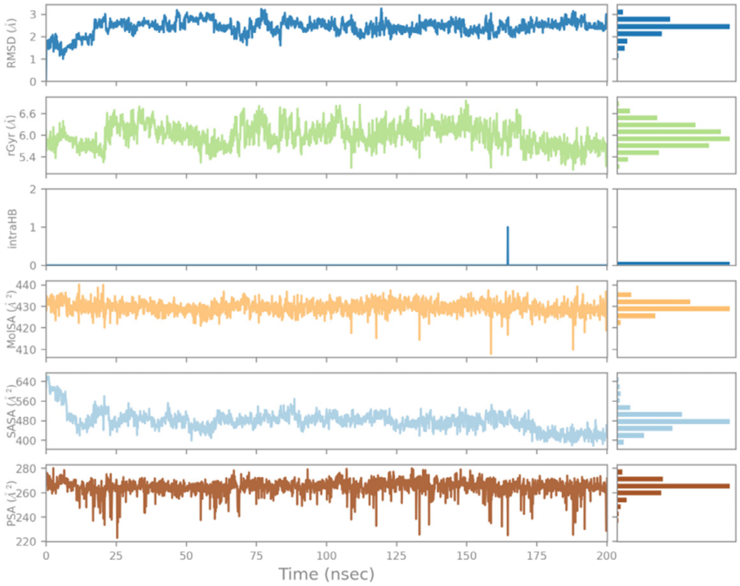
RMSD (Å)
0
1
2
3
rGyr (Å)
5.4
6.0
6.6
intraHB
0
1
2
MolSA (Å²)
410
420
430
440
SASA (Å²)
400
480
560
640
PSA (Å²)
220
240
260
280
0
25
50
75
100
125
150
175
200
Time (nsec)

B

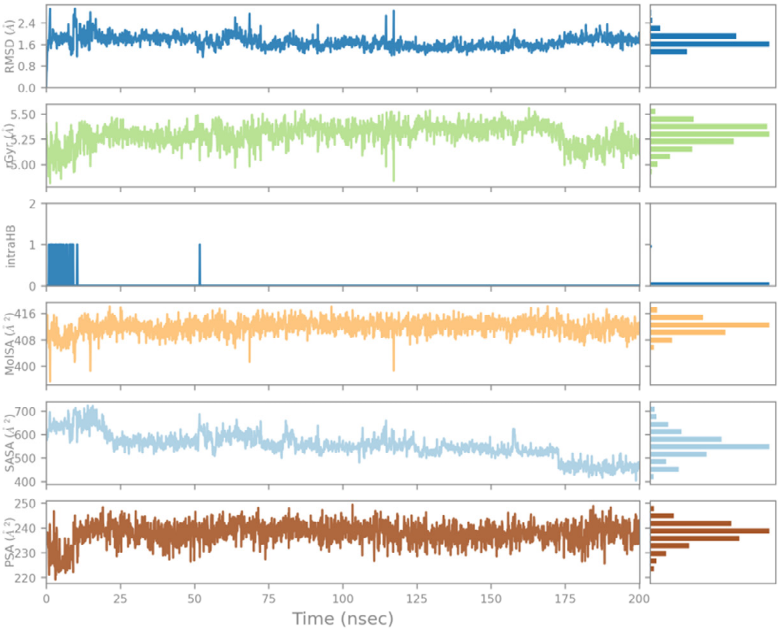

C

Figure S2. Ligand properties of the top three compounds complexed with HDAC6 (PDB ID: 6PYE) during 200 ns molecular dynamics simulation using Desmond software. (A) compound A, (B) compound B, and (C) compound C.

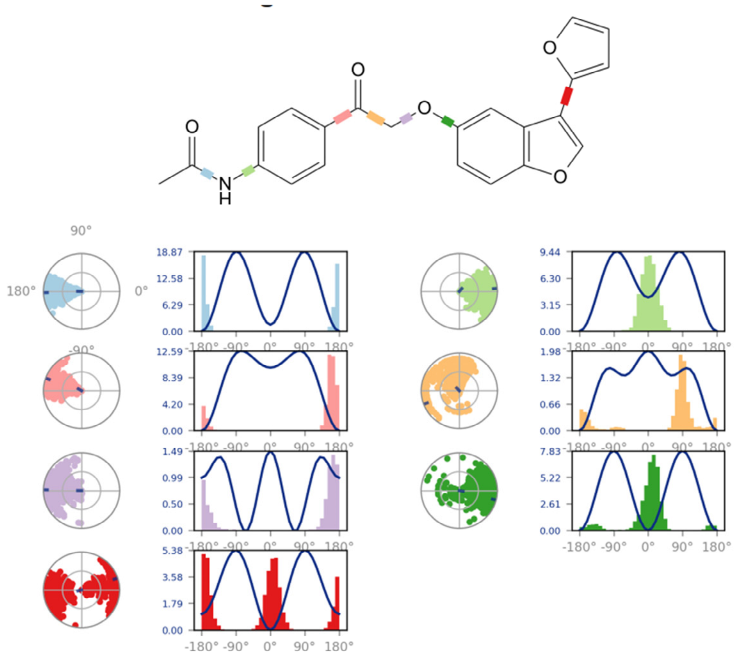

A

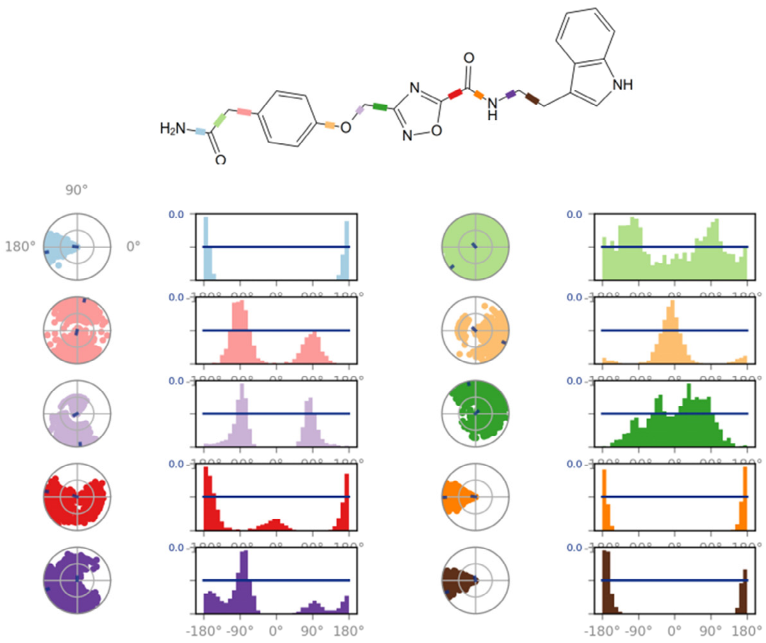

B

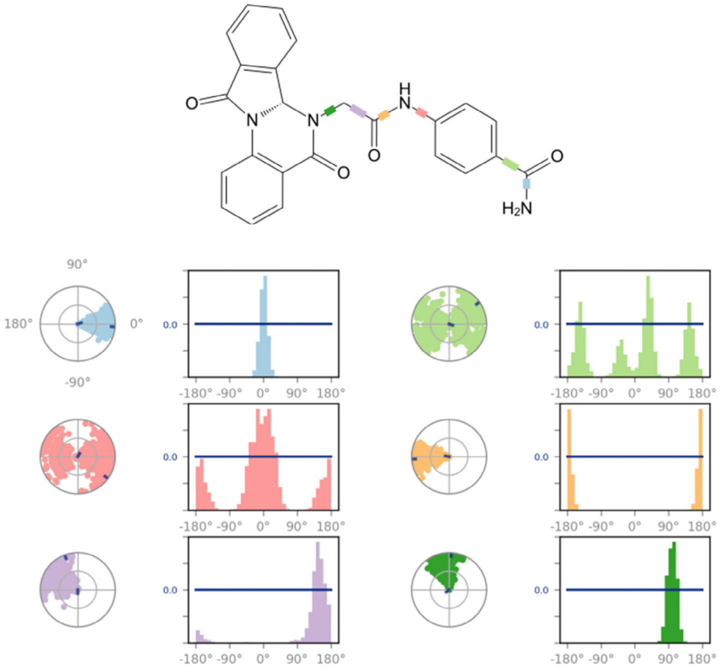

C

Figure S3. Ligand torsion Profile of the top three compounds complexed with HDAC6 (PDB ID: 6PYE) during 200 ns molecular dynamics simulation using Desmond software. The top panel shows the 2d schematic of a ligand with color-coded rotatable bonds. Each rotatable bond torsion is accompanied by a dial plot and bar plots of the same color. (A) compound A, (B) compound B, and (C) compound C.
